# The Second Allele: A Key to Understanding the Timing of Sporadic and Hereditary Colorectal Tumorigenesis

**DOI:** 10.3390/genes12101515

**Published:** 2021-09-26

**Authors:** Mohammed Ali Abbass, Brandie Leach, James Michael Church

**Affiliations:** 1 Northwestern Medicine Digestive Health Center, Chicago, IL 60611, USA; Mohammed.abbass@nm.org; 2 Invitae, San Francisco, CA 94103, USA; leachb@ccf.org; 3 Division of Colorectal Surgery, Columbia University Medical Center, New York, NY 10032, USA

**Keywords:** colorectal cancer, hereditary syndromes, molecular genetics, tumor suppression gene, second hit, allelic loss, methylation

## Abstract

Our understanding of the molecular basis of colorectal neoplasia is derived from Mendelian genetics, with tumor suppressor genes contributing more to the deregulation of growth than oncogenes. In patients with hereditary syndromes, expression of one allele of a key tumor suppressor gene is absent at birth. The loss of the expression of the second allele precipitates tumorigenesis. However, there are multiple ways in which the expression of the second allele of a tumor suppressor gene is lost. Here, we review these ways and their possible effect on phenotype.

## 1. Introduction

Cancers are caused by an accumulation of pathogenic variants in tumor suppressor genes and proto-oncogenes, conferring on the affected clone of cells an ability to escape normal regulation of growth and differentiation. Oncogenes contribute to the tumorigenic drive when they acquire a pathogenic activating variant in one allele. Tumor suppressor genes are different. If one allele is dysfunctional due to a pathogenic variant, the second allele, which is usually functioning normally (wild type), produces enough protein to maintain control over cell growth. For abnormalities in a tumor suppressor gene to cause disease, Knudsen’s “2 hit” theory states that both alleles must be inactivated [1]. This is the basis for the genetic model of tumorigenesis.

The origins of colorectal carcinoma have been open to genetic analysis for decades, because of the accessibility of premalignant lesions to colonoscopy. In 1975 Muto et al. described the development of increasingly severe dysplasia and increasing villous architecture in a small fraction of colorectal adenomas that suggested progressive destabilization of growth over many years [2]. Thirteen years later, Vogelstein published a series of chromosomal deletions that, along with “ras-gene mutations”, were associated with this progressive histological dysplasia in the colon and rectum [3]. The adenoma-carcinoma sequence previously described by Muto et al. [2]. now had a genetic explanation. Subsequently, details of the tumor suppressor genes and oncogenes involved in colorectal cancer emerged, with the identification of *APC*, *KRAS*, *SMAD4* and *p53* among others [4]. In more recent research, Vogelstein and his group suggested a relationship between cancer risk and the rate of division of stem cells in an epithelium [5]. The exceptionally high rate of cell division of colorectal stem cells predisposes their DNA to pathogenic variants, and is reflected in the high risk of colorectal cancer in the population. While genetic analysis of colorectal cancers shows pathogenic variants in large numbers of genes, the tumorigenesis is pushed by only a few “driver” genes [6]. In Vogelstein’s modeling, loss or inappropriate gain of function of three driver genes is all that is necessary for an affected clone to accumulate the required instability to ultimately become malignant.

The time required for sporadic colorectal neoplasia to develop is relatively long because multiple biologic systems guard the fidelity of DNA replication. Sporadic adenomas usually start to appear in the fifth and sixth decades of life, with progression from adenoma to carcinoma taking an average of 10 to 15 years [7]. This reflects the time needed for a clone of cells to lose expression of both alleles of at least two tumor suppressor driver genes, and gain an activating variant of one allele of at least one oncogene. That the process accelerates can be assumed from the 50 years or so it takes for one small sporadic adenoma to develop compared to the 10 years it takes for that small adenoma to become malignant. It is likely that the loss of function of the first driver gene facilitates loss of function of a second, which then facilitates the third. The whole process of colorectal tumorigenesis is accelerated in the hereditary syndromes of the disease, because patients are born with a pathogenic variant in a tumor suppressor gene in their germline. Loss of function of that gene occurs when a sporadic event causes loss of function of the wild type allele (the “second” allele), and consideration of the fate of this second allele can help in understanding the biology of colorectal carcinogenesis. The purpose of this article is to review the ways in which loss of the wild type allele might introduce variability into the phenotype of various syndromes of hereditary colorectal cancer. 

## 2. Hereditary Colorectal Cancer Syndromes as Models

### 2.1. Autosomal Dominant Syndromes

Of all the syndromes of hereditary colorectal cancer, familial adenomatous polyposis (FAP) is the best model for considering the effects of a germline pathogenic variant in a tumor suppressor gene. The gene involved, *APC*, is the key driver gene for both sporadic and hereditary colorectal tumorigenesis. Loss of APC leads to constantly active wnt signaling, chromosomal instability with loss of heterozygosity of multiple genes, and produces cytologic dysplasia [8]. Patients with FAP are born with a pathogenic variant affecting one *APC* allele in every cell in their body but are initially protected from the effects of this variant by the wild type allele. The first sporadic variant in *APC* that occurs in these patients is actually in the second genetic event (or “hit”, as described by Knudsen) striking the gene. As 90% of FAP patients have colorectal adenomas by age 18, it is likely that one of the effects of the germline variant is to encourage loss of expression of the second allele. In fact, the set up in FAP, with a germline pathogenic variant in a key tumor suppressor gene, occurring in an organ with rapid stem cell division, and exposed to all the carcinogens in the diet and the microbiome, creates a perfect storm of colorectal tumorigenesis. This is different to the scenario in fibroblasts. Desmoid tumors are a benign proliferation of fibroblasts, and are the second most common cause of death in patients with FAP [9]. Fibroblasts are unlike colonocytes in being mesenchymal-derived, with slower cell turnover, and are exposed to a completely different environment to that of colonocytes. In FAP, patients are born with a germline pathogenic *APC* variant in every fibroblast in their body. The wild type allele presumably prevents desmoid formation in most patients until its expression is lost, most likely due to trauma (surgical or otherwise).

### 2.2. Biallelic Germline Variants

Sometimes, patients are born with pathogenic variants in both alleles of a tumor suppressor gene. This is seen with Lynch syndrome genes in a syndrome called constitutional mismatch repair deficiency (CMMRD), which features very early onset of brain tumors, neurofibromatosis, colorectal cancers, hematological malignancies and sarcomas [10,11]. The different spectrum of tumors compared to that seen in classical Lynch syndrome likely reflects a lower level of sporadic loss of the second allele in CMMRD-specific tumor tissues, possibly related to slower stem cell divisions, different environments, and different cell origins compared to the classical Lynch organs. When the second allele is lost at conception the tissues that are particularly vulnerable to defective mismatch repair become obvious: brain, intestinal epithelium, blood, connective tissue. In addition to the unique spectrum of tumors and the young age of onset, CMMRD is nearly fully penetrant in affected individuals. Penetrance in the parents, each with one germline pathogenic variant in a mismatch repair gene, is often low, providing further evidence of the relative stability of the second allele. The phenomenon of CMMRD suggests that one of the influences determining penetrance in families with Lynch syndrome may be the ease and the mechanism with which the second allele of the variant mismatch repair gene is lost.

*MSH3* is a mismatch repair gene that dimerizes with *MSH2* and is involved in the repair of di and tetranucleotide mismatches [12]. While monoallelic germline *MSH3* pathogenic variants do not cause Lynch syndrome, again possibly because of the stability of the wild-type allele, biallelic *MSH3* pathogenic variants present clinically not with the typical CMMRD phenotype involving multiple organs, but just as adenomatous polyposis characterized by EMAST (elevated microsatellite alterations at selected tetranucleotide repeats) in the polyps [13].

### 2.3. Other Recessive Polyposes

There are two other recessively inherited syndromes of colorectal cancer, both related to loss of base excision repair. The more common and more clinically obvious syndrome, is *MUTYH*- associated polyposis (MAP). In patients carrying a pathogenic *MUTYH* variant in a single allele, there is a debatable but small increase in colorectal cancer risk, estimated at twice the average population risk [14]. This risk is relatively low because the second allele is quite stable and sporadic loss is uncommon. However, when pathogenic variants are inherited in both alleles, there is adenomatous polyposis with a much higher risk of cancer [15]. Tumorigenesis occurs through GC: TA transversions caused by loss of base excision repair and the effect of these transversions on downstream driver genes such as *APC* [14]. MAP is a milder version of classic FAP, with a few differences in phenotype. As with any hereditary loss of DNA repair, both alleles of a downstream tumor suppressor gene can be affected simultaneously, accelerating tumorigenesis. The other recessively inherited syndrome of colorectal polyposis is *NTHL1*-associated polyposis, due to biallelic pathogenic variants in *NTHL1.* This also leads to a failure of base excision repair with C:G to T:A transitions [16].

### 2.4. Sessile Serrated Polyposis and Epigenetic Loss of the Second Allele

Serrated polyposis is another disease where the fate of the second allele is likely to affect phenotype and management. It has recently been redefined clinically by the World Health Organization as either Type 1 (≥5 Serrated lesions/polyps proximal to the rectum, all being ≥5 mm in size, with ≥2 being ≥10 mm in size), or Type 2 (>20 Serrated lesions/polyps of any size distributed throughout the large bowel, with ≥5 being proximal to the rectum) [17]. Although the definitions do not mention the details of histology or biology, the most homogenous form of this disease is Type 1, where the polyps are usually right sided, sessile serrated lesions (SSL) that are associated with a particular pattern of DNA hypermethylation known as CIMP (CpG Island Methylator Phenotype). It is unlikely that serrated polyposis is caused by a single germline pathogenic variant in any gene [18]. The most likely cause remains a high level of sporadic *BRAF* variants, possibly related to an environmental factor such as smoking. The phenotype is multiple sessile serrated lesions and methylated adenomas, with a high risk of interval and metachronous cancers. The progression of serrated and adenomatous lesions toward cancer seen in these patients can be rapid. Interval cancers can develop in a year. This aggressive tumorigenesis may be due to synchronous hypermethylation of the promoter region of both alleles of a tumor suppressor gene causing instant loss of expression. Furthermore, methylated adenomas are more aggressive than adenomas in colons without CIMP, suggesting that the hypermethylation abrogates gene expression in both alleles of tumor suppressor genes in these polyps too [19].

## 3. Loss of Expression of the Second Allele as a Cause of Variability in Phenotype

Patients affected by syndromes of hereditary colorectal cancer show considerable variability in phenotype, both between and within families. Variability between families is largely due to differences in genotype but may also involve differences in modifier genes, environmental exposures, or comorbid conditions and their treatment. Within families, the genotype of the syndrome is uniform and variations in penetrance, age of onset, tumor spectrum and the severity of the neoplasia are more difficult to understand [20]. It is worth considering the different ways in which expression of the second allele can be lost, as these may influence the phenotype of hereditary syndromes both between and within families. 

### 3.1. Development and Timing of a Sporadic Pathogenic Variant Affecting the Second (Wild Type) Allele

The development of a sporadic pathogenic variant in the second allele may be influenced by variable environmental exposures such as smoking, exercise and diet. The high rate of stem cell division in the intestine makes this organ particularly susceptible to the development of stochastic variants in the second allele that may be pathogenic.

### 3.2. Hypermethylation

Promoter methylation is an epigenetic phenomenon that controls gene expression. Increased levels of methylation decrease gene expression and hypermethylation can abrogate it. Because hypermethylation is a generalized phenomenon it can potentially affect both alleles of any susceptible gene [21,22]. There is a strong relationship between smoking, pathogenic variants in *BRAF*, and CIMP [22,23]. This introduces an easily measured source of phenotypic variation in all hereditary colorectal cancer syndromes.

### 3.3. Gene Conversion

Gene conversion is a process by which a DNA sequence on one allele replaces an equivalent but different sequence on the other allele, meaning that the alleles are now identical. Zhang et al. noted that somatic mutations in the second allele of patients with a germline pathogenic variant in a mismatch repair gene were identical to the germline variant [24]. They suggested that loss of the second allele occurred mainly due to gene conversion. The effect of such a phenomenon on phenotype has not been explored.

### 3.4. Dominant Negative Effect

A dominant negative effect occurs when the truncated protein produced by a mutated allele interferes with the function of the full-length protein from the wild type allele. *APC* is a good example of this, where variants in the “mutation cluster region” including codon 1309 produce profuse polyposis, but variants at the 3′ and 5′ end of the gene are associated with attenuated polyposis. The truncated protein produced by a gene with a 1309 variant inhibits wild type APC and causes a severe colorectal phenotype. Variants at the 3′ and 5′ ends of the gene produce either a very small protein or a protein of almost normal length, allowing wild type APC to function almost normally and producing attenuated polyposis [25,26].

### 3.5. Haplotype Insufficiency

Haplotype insufficiency is all about the “dose “of the protein produced by the combination of the variant allele and the wild type allele. If the dose is too low for normal function, haplotype insufficiency can occur [27,28,29]. Sometimes a wild type allele is not fully expressed, producing a reduced “dose “of the relevant protein that can cause functional effects when the other allele is affected by a pathogenic variant in the germline.

### 3.6. Microsatellite Instability and Mutational Biases

When there is deficient DNA mismatch repair in a clone of cells, there is a potential for cells to develop pathogenic variants in genes that contain a microsatellite. The effect of pathogenic variants in mismatch repair genes seems to vary according to the particular mismatch repair gene that is inactivated and the particular microsatellite involved [30]. In addition, some mismatch repair genes (*MSH6*, *MSH3*, *PMS2* and *MLH3*) contain mononucleotide microsatellites, making mismatch repair deficient clones vulnerable to worsening mutagenesis if there is already microsatellite instability due to pathogenic variants in *MLH1* or *MSH2*.

### 3.7. The Effect of the Microbiome

The human microbiome is marked by considerable inter-individual variability in organisms and in its own genome, and so is a potential source of variation in colorectal tumorigenesis [31]. While associations between aspects of the microbiome and colorectal tumorigenesis have been demonstrated in a general sense, a recent report suggests that a genotoxic pks+ *E. coli* may cause specific sequence changes in the DNA [32,33]. This opens the door to considering the microbiome as a further cause of variability in the loss of the wild type allele.

## 4. Discussion and Summary

In summary, the variability in phenotype seen so commonly in patients with both sporadic and hereditary colorectal cancer has multiple potential causes. Loss of expression of the “wild type” or second allele of tumor suppressor genes is the precipitating event in tumorigenesis, and is potentially related to multiple different genetic events. Variation in the stability of the second allele, and the mechanism of its loss in cells already affected by a constitutional pathogenic variant in one allele, are likely to influence both the timing and the severity of tumor formation in hereditary syndromes of colorectal cancer. This variability may be clinically significant in syndromes subject to it, as the pattern of disease in the family cannot be used to predict the course in an individual. Surveillance strategies should take this in to account by assuming risk to be high initially and lengthening intervals between examinations as this is shown to be safe. The effect of chemopreventive drugs on abnormal cell division may differ according to the mechanism of the genetic instability [34,35]. This would encourage tumor sequencing, paying attention to the mechanism of loss of the second allele and using genetic signatures as information to guide therapy. This approach is an important topic for future work.

## Data Availability

Not applicable.

## References

[1] Knudson A.G. (1971). Mutation and cancer: Statistical study of retinoblastoma. Proc. Natl. Acad. Sci. USA.

[2] Muto T., Bussey H.J., Morson B.C. (1975). The evolution of cancer of the colon and rectum. Cancer.

[3] Vogelstein B., Fearon E.R., Hamilton S.R., Kern S.E., Preisinger A.C., Leppert M., Nakamura Y., White R., Smits A.M., Bos J.L. (1988). Genetic alterations during colorectal-tumor development. N. Engl. J. Med..

[4] Cho K., Vogelstein B. (1992). Genetic alterations in the adenoma–carcinoma sequence. Cancer.

[5] Tomasetti C., Vogelstein B. (2015). Variation in cancer risk among tissues can be explained by the number of stem cell divisions. Science.

[6] Tomasetti C., Marchionni L., Nowak M.A., Parmigiani G., Vogelstein B. (2015). Only three driver gene mutations are required for the development of lung and colorectal cancers. Proc. Natl. Acad. Sci. USA.

[7] Winawer S.J. (1999). Natural history of colorectal cancer. Am. J. Med..

[8] Hankey W., Frankel W.L., Groden J. (2018). Functions of the APC tumor suppressor protein dependent and independent of canonical WNT signaling: Implications for therapeutic targeting. Cancer Metastasis Rev..

[9] De Marchis M.L., Tonelli F., Quaresmini D., Lovero D., Della-Morte D., Silvestris F., Guadagni F., Palmirotta R. (2017). Desmoid Tumors in Familial Adenomatous Polyposis. Anticancer Res..

[10] Bakry D., Aronson M., Durno C., Rimawi H., Farah R., Alharbi Q.K., Alharbi M., Shamvil A., Ben-Shachar S., Mistry M. (2014). Genetic and clinical determinants of constitutional mismatch repair deficiency syndrome: Report from the constitutional mismatch repair deficiency consortium. Eur. J. Cancer.

[11] Lavoine N., Colas C., Muleris M., Bodo S., Duval A., Entz-Werle N., Coulet F., Cabaret O., Andreiuolo F., Charpy C. (2015). Constitutional mismatch repair deficiency syn-drome: Clinical description in a French cohort. J. Med. Genet..

[12] Adam R., Spier I., Zhao B., Kloth M., Marquez J., Hinrichsen I., Kirfel J., Tafazzoli A., Horpaopan S. (2016). Exome Sequencing Identifies Biallelic MSH3 Germline Mutations as a Recessive Subtype of Colorectal Adenomatous Polyposis. Am. J. Hum. Genet..

[13] Carethers J.M., Koi M., Tseng-Rogenski S.S. (2015). EMAST is a Form of Microsatellite Instability That is Initiated by Inflammation and Modulates Colorectal Cancer Progression. Genes.

[14] Jenkins M., Croitoru M.E., Monga N., Cleary S., Cotterchio M., Hopper J.L., Gallinger S. (2006). Risk of Colorectal Cancer in Monoallelic and Biallelic Carriers of MYH Mutations: A Population-Based Case-Family Study. Cancer Epidemiol. Biomark. Prev..

[15] Al Tassan N., Chmiel N.H., Maynard J., Fleming N., Livingston A.L., Williams G.T., Hodges A., Davies D.R., David S.S., Sampson J.R. (2002). Inherited variants of MYH associated with somatic G:C→T:A mutations in colorectal tumors. Nat. Genet..

[16] Weren R.D., Ligtenberg M.J., van Kessel A.G., de Voer R.M., Hoogerbrugge N., Kuiper R.P. (2018). NTHL1 and MUTYH polyposis syndromes: Two sides of the same coin?. J. Pathol..

[17] Dekker E., Bleijenberg A., Balaguer F., Ijspeert J.E., Pellisé M., Carballal S., Rivero L., Latchford A. (2020). Update on the World Health Organization Criteria for Diagnosis of Serrated Polyposis Syndrome. Gastroenterology.

[18] Cauley C.E., Hassab T.H., Feinberg A., Church J. (2020). Sessile Serrated Polyposis: Not an Inherited Syndrome?. Dis. Colon Rectum.

[19] Pai R.K., Mackinnon A.C., Joseph L., Noffsinger A., Hart J. (2010). Identification of Histologically Distinct Conventional Adenomas that Arise Predominately in Patients with Sessile Serrated Adenomas. Am. J. Surg. Pathol..

[20] Brensinger J.D., Laken S.J., Luce M.C., Powell S.M., Vance G.H., Ahnen D.J., Petersen G.M., Hamilton S.R., Giardiello F.M. (1998). Variable phenotype of familial adenomatous polyposis in pedigrees with 3′ mutation in the APC gene. Gut.

[21] Peltomäki P. (2012). Mutations and epimutations in the origin of cancer. Exp. Cell Res..

[22] Noreen F., Küng T., Tornillo L., Parker H., Silva M., Weis S., Marra G., Rad R., Truninger K., Schär P. (2019). DNA methylation instability by BRAF-mediated TET silencing and lifestyle-exposure divides colon cancer pathways. Clin. Epigenet..

[23] Samowitz W.S., Albertsen H., Sweeney C., Herrick J., Caan B., Anderson K.E., Wolff R.K., Slattery M.L. (2006). Association of Smoking, CpG Island Methylator Phenotype, and V600E BRAF Mutations in Colon Cancer. J. Natl. Cancer Inst..

[24] Zhang J., Lindroos A., Ollila S., Russell A., Marra G., Mueller H., Peltomaki P., Plasilova M., Heinimann K. (2006). Gene conversion is a frequent mechanism of inactivation of the wild-type allele in cancers from MLH1/MSH2 deletion carriers. Cancer Res..

[25] Dihlmann S., Gebert J., Siermann A., Herfarth C., von Knebel Doeberitz M. (1999). Dominant negative effect of the APC1309 muta-tion: A possible explanation for genotype-phenotype correlations in familial adenomatous polyposis. Cancer Res..

[26] Payne S.R., Kemp C.J. (2005). Tumor suppressor genetics. Carcinogenesis.

[27] Inoue K., Fry E.A. (2017). Haploinsufficient tumor suppressor genes. Adv. Med. Boil..

[28] Venesio T., Balsamo A., Rondo-Spaudo M., Varesco L., Risio M., Ranzani G.N. (2003). APC haploinsufficiency, but not CTNNB1 or CDH1 gene mutations, accounts for a fraction of familial adenomatous polyposis patients without APC truncating mutations. Lab Investig..

[29] Gaspar C., Fodde R. (2004). APC dosage effects in tumorigenesis and stem cell differentiation. Int. J. Dev. Biol..

[30] Shah S.N., Hile S.E., Eckert K.A. (2010). Defective mismatch repair, microsatellite mutation bias, and variability in clinical cancer phenotypes. Cancer Res..

[31] Lloyd-Price J., Abu-Ali G., Huttenhower C. (2016). The healthy human microbiome. Genome Med..

[32] Allen J., Sears C.L. (2019). Impact of the gut microbiome on the genome and epigenome of colon epithelial cells: Contributions to colorectal cancer development. Genome Med..

[33] Purcell R.V., Visnovska M., Biggs P.J., Schmeier S., Frizelle F.A. (2017). Distinct gut microbiome patterns associate with consensus molecular subtypes of colorectal cancer. Sci. Rep..

[34] O’Brien C., Giannakis M., Artursson P., Nygren P., Cheong I., Sjöblom T. (2020). Exploiting loss of heterozygosity for allele-selective colorectal cancer chemotherapy. Nat. Commun..

[35] Rendo V., Kundu S., Rameika N., Ljungström V., Svensson R., Palin K. (2020). Defining eligible patients for allele-selective chemotherapies targeting NAT2 in colorectal cancer. Sci. Rep..

